# Thromboelastographic Assessment of the Differential Sensitivity of Three Contact Activators to Coagulation Factor Deficiencies in Hemocell Environments

**DOI:** 10.3390/diagnostics16193217

**Published:** 2026-10-04

**Authors:** Weiguo Liang, Mao Xia, Junpo Li, Fuyang Li, Bidou Wang, Haixuan Sun

**Affiliations:** 1 School of Biomedical Engineering (Suzhou), University of Science and Technology of China, Hefei 230026, China; liangwg@sibet.ac.cn; 2 Suzhou Institute of Biomedical Engineering and Technology, Chinese Academy of Sciences, Suzhou 215000, China; lijp@sibet.ac.cn (J.L.); lify@sibet.ac.cn (F.L.); 3 Affiliated Hospital of Nantong University, Nantong 226000, China; xiamao0126@163.com

**Keywords:** thromboelastography (TEG), contract activator, reagent sensitivity, coagulation factor deficiencies, reconstituted whole-blood sample

## Abstract

**Background:** Thromboelastography (TEG) assesses global coagulation, but its sensitivity for factor deficiencies is reportedly low and may depend on the contact activator. However, the differential TEG sensitivity of contact activators for individual factor deficiencies in whole blood containing platelets and red blood cells remains unclear. This study assessed the relative sensitivity of three contact activators for detecting single coagulation factor deficiencies in reconstituted whole-blood samples using TEG. **Methods**: Reconstituted whole-blood samples with graded activities of FII, FV, FVIII, FIX, FX, FXI, and FXII were prepared by mixing platelets, plasma, and red blood cells, and analyzed by TEG using kaolin-, ellagic acid-, and colloidal silica-based activators (KL, EA and CS). Relative sensitivity was assessed via: (1) the factor activity at which R time first showed a statistically significant prolongation; (2) a novel metric, Sensitivity for Detecting Factor Deficiencies (SDFD), defined as the normalized relative change in R time, enabling comparison without reference ranges. **Results**: KL showed the broadest and highest sensitivity, detecting significant prolongation at the mildest deficiency levels for most factors. EA showed intermediate-to-high sensitivity and the highest sensitivity for FXII. CS was the least sensitive except for FV. Notably, the activity threshold of KL for exceeding the reference range was substantially lower than that for statistical significance. **Conclusions**: The sensitivity of TEG to coagulation factor deficiencies is dependent on the contact activator. KL showed the highest overall sensitivity to factor deficiencies, while EA showed the highest sensitivity for FXII deficiency under the conditions of this study. R time of KL can be significantly prolonged yet still stay within the reference range. This mismatch calls for more sensitive TEG reagents and stricter ranges.

## 1. Introduction

Coagulation factor deficiencies can be associated with a bleeding phenotype ranging from mild to severe; there is a strong association between coagulation factor activity level and clinical bleeding severity for factor (F) I, FX, FXIII, FV, and FVIII deficiencies [1]. The activated partial thromboplastin time (APTT) and the prothrombin time (PT) are the most widely employed assays for detecting coagulation factor deficiencies and managing rare coagulation factor deficiencies, such as hemophilia [2,3]. However, the APTT and PT lack platelets and only measure the time from coagulation initiation to the initial fibrin clot formation—an endpoint that reflects only 5% of thrombin generation in the overall coagulation process [4]. In contrast to these plasma-based assays, thromboelastography (TEG) assesses hemostasis in whole blood, capturing not only the enzymatic coagulation cascade but also the contributions of platelets, fibrinogen, and the fibrinolytic system, thereby providing a physiologically more comprehensive evaluation of overall hemostatic function [5,6].

Thromboelastography (TEG) is a point-of-care testing technique that monitors the viscoelastic changes of whole blood during coagulation. The data extracted from the TEG curve include reaction time (R time), coagulation time (K time), angle and maximum amplitude (MA). It provides a dynamic and comprehensive assessment of the overall hemostatic function by tracing the entire process from initial fibrin formation and clot stabilization to eventual fibrinolysis [5]. TEG has been widely used in various clinical settings [7,8,9,10,11,12]. In the context of coagulation factor deficiency screening, TEG is primarily applied for detecting factor deficiencies in trauma patients, identifying specific factor deficits (e.g., FXIII), assessing hemophilia phenotypes, and monitoring anticoagulant therapy [11,13,14,15]. However, current evidence indicates that TEG has limited and inconsistent sensitivity for detecting coagulation factor deficiencies, which restricts its broader application in this specific screening role [11,16,17,18].

A review article has indicated that alterations in thromboelastography detection reagents can lead to changes in assay sensitivity, highlighting that the choice of reagent is critical for the sensitivity in detecting coagulation factor deficiencies [19]. In conventional coagulation assays such as APTT, the type of activator used influences reagent sensitivity [20]. Currently, the intrinsic pathway activator employed in TEG is kaolin, whereas two other viscoelastic whole-blood assay systems, rotational thromboelastometry (ROTEM) and Sonoclot, utilize ellagic acid and silica as their respective activators. Differences in the sensitivity for detecting coagulation factor deficiencies among various activators have been investigated in traditional coagulation methods [20,21,22,23,24,25]. However, those studies were conducted using plasma samples, which differ substantially from the whole-blood specimens employed in TEG analysis. Critically, whether the activator-dependent sensitivity differences observed in plasma also hold true in whole-blood viscoelastic assays such as TEG remains unclear. Nielsen et al. investigated the effects of coagulation factor deficiencies on plasma coagulation kinetics using thromboelastography and compared the differences between celite and tissue factor in the context of factor deficiencies [26]. Moreover, this study was still based on plasma samples, and the comparison was made between celite (an intrinsic pathway activator) and tissue factor (an extrinsic pathway activator) under conditions of coagulation factor activity less than 1%. However, both platelets and RBCs contribute to coagulation and influence TEG parameters directly or indirectly. Approximately 80% of MA has been attributed to platelet function, with the remaining 20% attributed to fibrin [27]. Procoagulant platelets expose phosphatidylserine, which supports tenase and prothrombinase assembly and enhances thrombin generation, and may thereby influence R time [28]. RBCs can also contribute to thrombin generation and clot formation through phosphatidylserine exposure, modulation of platelet reactivity, and effects on fibrin structure and mechanics, which may in turn influence TEG parameters [29,30]. Consequently, whether the three contact activators (kaolin, ellagic acid, and silica) differ in their TEG sensitivity for detecting deficiencies of different individual coagulation factors, and whether such differences vary across factor activity levels, in the presence of cellular components—platelets and red blood cells—remains to be systematically evaluated.

In this study, we constituted in vitro whole blood models with varying coagulation factor activities. Using reconstituted whole-blood samples, we employed thromboelastography (TEG) to compare the sensitivity of three activators (kaolin, ellagic acid, and silica) in detecting coagulation factor deficiencies in the presence of platelets and red blood cells, thereby providing a reference for activator selection in developing high-sensitivity TEG reagents.

## 2. Materials and Methods

### 2.1. Commercial Plasma

Clotting Factor Deficient Plasma and Standard Human Plasma (lyophilized) were purchased from Siemens Healthcare Diagnostics Products GmbH (Marburg, Germany). Clotting factor-deficient FII, FV, FVIII, FIX, FX, FXI and FXII plasmas contained corresponding single factor activity ≤ 1%, fibrinogen ≥ 1 g/L, and the residual coagulation factor activity of 50% or more. Standard Human Plasma with coagulation factor activity of 91%, 85%, 84%, 84%, 92%, 92%, and 101% of FII, FV, FVIII, FIX, FX, FXI and FXII, respectively, and 2.451 g/L of fibrinogen, were used. The plasma was reconstituted in deionized water prior to use.

### 2.2. Chemical Reagents

Kaolin, LUDOX^®^ AM colloidal silica, potassium chloride and sodium chloride were from Sigma-Aldrich^®^ (St. Louis, MO, USA). Polyethylene glycol 4000 and betaine were obtained from Sinopharm Group Co., Ltd. (Shanghai, China). Ethylene glycol-bis(2-aminoethylether)-N, N, N′, N′-tetraacetic acid (EGTA), hydroxyethyl piperazine ethanesulfonic acid (Hepes), imidazole, ellagic acid, vitamin B6 hydrochloride and sodium nitroprusside were produced by Shanghai Aladdin Biochemical Technology Co., Ltd. (Shanghai, China).

### 2.3. Preparation of TEG Test Activators (Reagents)

Kaolin-based activators (KL) were prepared by the following method, as used by WITEYE (Wuhan) Medical Technology Co., Ltd. (Wuhan, China). Briefly, Kaolin was dispersed in deionized water by vortex mixing for 10 min to a final concentration of 2% (*w*/*v*). Then, the kaolin suspension was diluted to 0.01% (*w*/*v*) using Hepes buffer (10 mM Hepes, 5 mM Betaine, 0.1% (*w*/*v*) Polyethylene glycol 4000, pH 7.4). Ellagic acid-based activators (EA) were produced using the following method. Ellagic acid was dispersed in deionized water by vortex mixing for 5 min to a final concentration of 0.6% (*w*/*v*), subsequently diluted to 0.0015% using the Hepes buffer. Silica-based activators (CS) were prepared by diluting the LUDOX ^®^ AM colloidal silica with the Hepes buffer to a final concentration of 0.15%. KL was a commercial clinical reagent used at its recommended working concentration, whereas EA and CS were in-house formulations calibrated against KL to achieve comparable functional activity.

For the same sample tested on TEG using EA and KL, CS and KL, the relative average deviations (RSD) of parameters R time, Angle, and MA were <15%, <10%, and <10%, respectively. Following the Product Technical Requirements of KL (WITEYE (Wuhan) Medical Technology Co., Ltd.), quality control samples (made in-house) were used to evaluate EA and CS. The acceptance criteria for repeatability and batch-to-batch variation were set at ≤15% for R, ≤10% for Angle, and ≤10% for MA, respectively.

### 2.4. Preparation of Platelets and Red Blood Cells

Platelet-rich plasma (PRP) was prepared by pooling PRP from multiple donors. Briefly, residual whole blood samples were obtained during routine clinical testing at the Affiliated Hospital of Nantong University, and the pooled PRP was separated from each donor by centrifugation at 200× *g* for 5 min. The individual PRP samples were then combined into a single pooled batch. PRP was centrifuged at 1000× *g* for 10 min. The platelet pellet was washed twice with a washing buffer (100 mM NaCl, 10 mM KCl, 10 mM EGTA, 10 mM imidazole, 40 µg/mL vitamin B6 hydrochloride, 6 µg/mL sodium nitroprusside, pH 6.8) modified from that of Wolkers, W. F. et al. [31]. The platelets were divided into two equal aliquots and resuspended in equal volumes of factor-deficient plasma and standard human plasma to prepare platelet-rich factor-deficient plasma (PRDP) and platelet-rich standard plasma (PRSP), respectively. PRSP was used for platelet counting, and the platelet count in the PRSP was required to be >1.2 × 10^8^/mL [32]. The platelets were counted using a Countstar Mira FL-S Automatic Cell Fluorescence Analyzer (Shanghai Ruiyu Biotech Co., Ltd., Shanghai, China). In each independent run, the platelets used for reconstituting whole blood samples for the same coagulation factor were derived from the same donor batch. Platelet function after washing and resuspension was assessed by measuring the MA value obtained with KL in reconstituted whole-blood samples at 100% factor activity (WITEYE T1000 Thromboelastograph; WITEYE (Wuhan) Medical Technology Co., Ltd.), using Standard Human Plasma as a reference control. MA primarily reflects platelet aggregation function.

Red blood cells were isolated from residual RBC suspensions remaining after blood typing tests for transfusion purposes at the same institution. For each independent run, residual RBC suspensions from multiple blood typing tests were pooled to prepare a single RBC batch. RBCs were centrifuged at 1000× *g* for 10 min and washed three times with 10 volumes of 0.9% NaCl solution. After washing, the supernatant was discarded, and the red blood cells were resuspended. For each coagulation factor, all reconstituted whole blood samples across its different activity levels were prepared using the same batch of RBC suspension derived from the same donor batch, with identical RBC addition volumes across all activity levels.

### 2.5. Reconstitution of Whole Blood

Within each independent run and for each coagulation factor, the reconstituted whole-blood samples used for testing all three activators were prepared using the same batch of plasma, platelet preparation, and RBC suspension.

A series of 7 reconstituted whole-blood samples with factor activities of 1–100% was obtained by mixing PRDP, PRSP, and RBCs according to the proportions shown in Table 1. In this study, the factor activity of reconstituted whole-blood samples was defined as equivalent to that of the plasma used for reconstitution.

### 2.6. Thromboelastography

All tests were performed on the WITEYE T1000 Thromboelastograph (WITEYE (Wuhan) Medical Technology Co., Ltd., Wuhan, China). Briefly, 0.5 mL of reconstituted whole-blood samples with different factor activities was added to a tube with KL, EA, or CS, respectively. Then, 340 μL of this blood was added to a test cup containing 20 μL of 0.2 M CaCl2. The experimental unit was each independent experimental run (*n* = 3), with samples independently prepared for each run.

The TEG curve provides several key parameters. R time (reaction time) is the latency period from the start of the assay until the clot amplitude reaches 2 mm. It reflects the initiation phase of coagulation and primarily represents coagulation factor activity. K time (kinetics time) is the interval from initial clot formation (2 mm) to a fixed clot firmness of 20 mm. It reflects the speed of clot strengthening. The α-angle (alpha angle) is the angle formed by the slope of the TEG tracing from the R time. It represents the kinetics of fibrin accumulation and cross-linking. MA (maximum amplitude) is the greatest amplitude of the TEG tracing. It reflects maximum clot strength, which is determined by platelet–fibrin interactions via glycoprotein IIb/IIIa receptors. The R time served as the main parameter in this study for evaluating the sensitivity of activators to coagulation factor deficiency. A prolonged R time suggests factor deficiency or the effect of anticoagulants [33]. The kaolin-activated R time reference range provided by the WITEYE T1000 is 3.9–8.3 min.

### 2.7. Calculation of Sensitivity for Detecting Factor Deficiencies(SDFD)

The Clinical and Laboratory Standards Institute (CLSI)-approved guidelines for activated partial thromboplastin time (APTT) recommend that an APTT reagent should produce a clear prolongation of the APTT to the upper limit of the normal reference range in plasmas containing less than 30% of factor VIII, IX, or XI (H47-A2, 2008).

According to the CLSI H47-A2 guideline for factor sensitivity assessment, each reagent requires the establishment of its own reference range. However, establishing reference ranges is time-consuming and region-dependent. Therefore, to evaluate the relative sensitivity of the three activators for detecting factor deficiencies without establishing reference ranges, sensitivity was calculated using the following method:

$$\mathrm{SDFD}=\frac{R_{i}-R_{\max}}{R_{\max}}\times 100\%$$

where *R_i_* is the R time of reconstituted whole-blood samples with varying factor activities (excluding *R_max_*), and *R_max_* is the R time of reconstituted whole-blood samples with the highest factor activity. SDFD was defined as the normalized relative change in R time and used as a relative analytical sensitivity metric to compare the three activators under the conditions of this study. A higher SDFD indicates greater relative sensitivity.

### 2.8. Statistical Analysis

Statistical analyses were performed with GraphPad Prism 10 (GraphPad Software, LLC, Boston, MA, USA). Data are presented as mean ± standard deviation. Data were analyzed using mixed-effects models with experimental batch as a random intercept. REML estimation was used. A one-way repeated-measures mixed model with Dunnett’s post-hoc test was used for factor activity-response comparisons within the same activator (R time of each activity level vs. the R time of maximum activity). A two-way repeated-measures mixed model with Tukey’s post-hoc test was used to compare SDFD among the three activators in two ways: (1) overall comparison, where the mean SDFD per activator was compared; and (2) comparison at each activity level, where the three activators were compared pairwise at each activity level. *p*-values were adjusted for multiple comparisons; adjusted *p* < 0.05 was considered significant. Mean differences with 95% confidence intervals are reported where appropriate.

## 3. Results

### 3.1. Performance Evaluation of EA and CS

The RSD values of R time, angle and MA between EA and KL, and between CS and KL, were <15%, <10%, and <10%, respectively. Both repeatability and batch-to-batch variation of EA and CS were within the required limits: ≤15% for R, ≤10% for Angle, and ≤10% for MA (Table 2).

### 3.2. Platelet Count and Function in Reconstituted Whole-Blood Samples

The PRSP platelet counts for the three independent runs were all >1.2 × 10^8^/mL (Table 3). The platelet count in the reconstituted whole-blood samples is approximately half of that in the PRSP.

Platelet function after washing and resuspension was assessed by comparing the MA value obtained with KL at 100% factor activity in reconstituted whole-blood samples with that of platelet-poor Standard Human Plasma (Table 4). The MA value in reconstituted whole-blood samples was substantially higher than that in standard plasma, supporting the retention of platelet contribution to clot strength after washing and resuspension.

### 3.3. Assessment and Comparison of the Sensitivity of Three Activators

**Figure 1:**
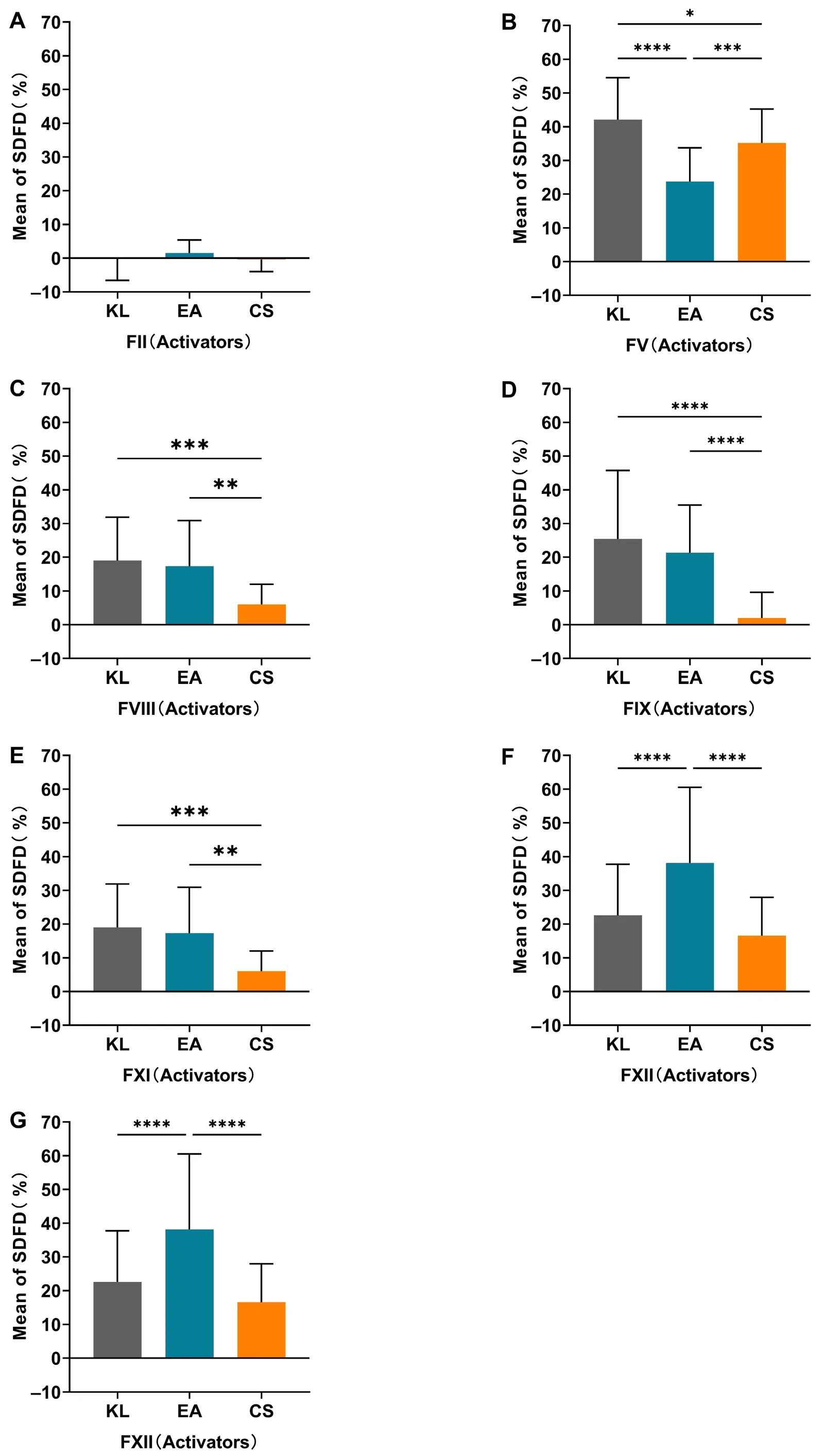
Overall Comparison of sensitivity for detecting factor deficiencies (SDFD) by mean SDFD values among three activators. (**A**) FII; (**B**) FV; (**C**) FVIII; (**D**) FIX; (**E**) FX; (**F**) FXI; (**G**) FXII. Data are mean ± SD (*n* = 3 independent experiments). A two-way repeated-measures mixed model with Tukey’s test was used. Mean SDFD value for each activator was derived from all activity levels. Only comparisons with significant differences are indicated by connecting lines and asterisks. Significance levels: * *p* < 0.05, ** *p* < 0.01, *** *p* < 0.001, **** *p* < 0.0001. Factor sensitivity increases with increasing SDFD value.

**Figure 2:**
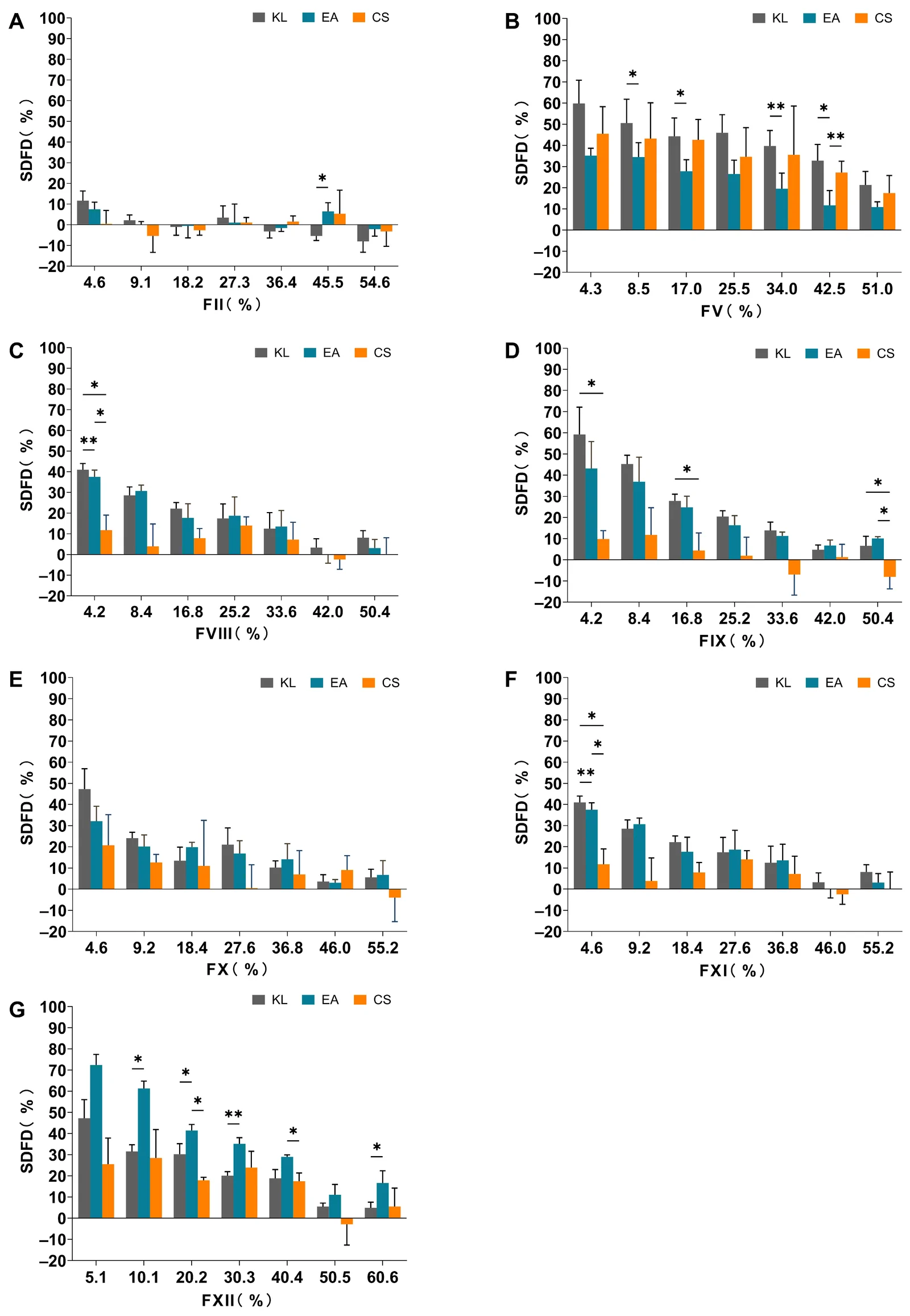
Comparisons at each factor activity level of sensitivity for detecting factor deficiencies (SDFD) values among three activators. (**A**) FII; (**B**) FV; (**C**) FVIII; (**D**) FIX; (**E**) FX; (**F**) FXI; (**G**) FXII. Data are mean ± SD (*n* = 3 independent experiments). A two-way repeated-measures mixed model with Tukey’s test was used. Only comparisons with significant differences are indicated by connecting lines and asterisks. Significance levels: * *p* < 0.05, ** *p* < 0.01. Factor sensitivity increases with increasing SDFD value.

**Figure 3:**
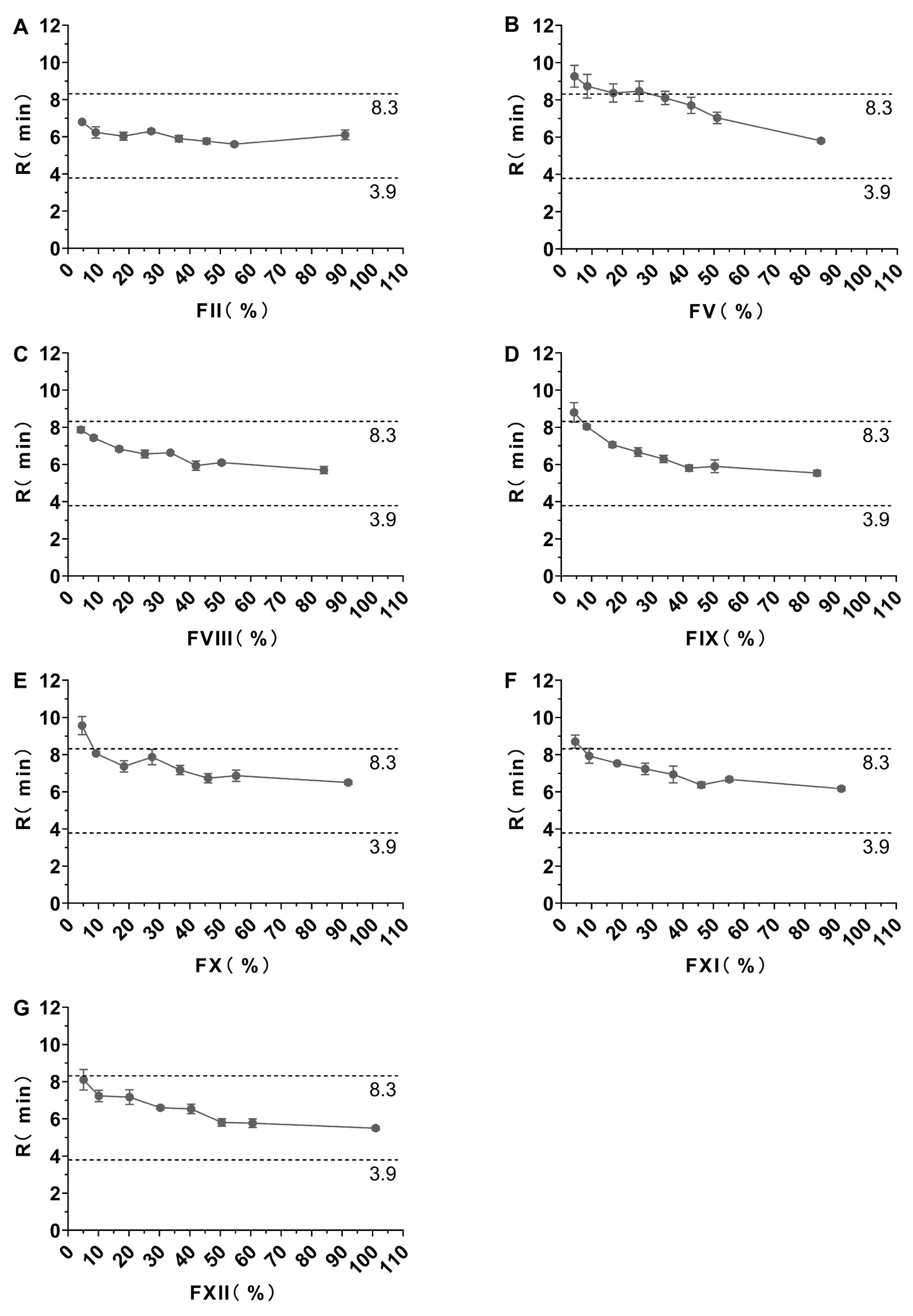
Evaluation of Kaolin-based activator (KL) factor sensitivity by R time. (**A**) FII; (**B**) FV; (**C**) FVIII; (**D**) FIX; (**E**) FX; (**F**) FXI; (**G**) FXII. Dotted lines indicate the reference range (3.9–8.3 min). Means ± SD (*n* = 3 independent experiments).

Our study aimed to systematically evaluate and compare the sensitivity of three distinct TEG contact activators—kaolin (KL), ellagic acid (EA), and colloidal silica (CS)—to single-factor deficiencies across the intrinsic and common pathways (Factors II, V, VIII, IX, X, XI, and XII). Sensitivity was primarily assessed by R time. This study employed two analytical approaches: first, determining the factor activity level at which a statistically significant prolongation of R time first occurred (Table 5); second, and more importantly, we applied a novel factor sensitivity calculation method (SDFD) to directly compare the sensitivity of the three activators, both overall (mean SDFD per activator) and at each individual activity level (Figure 1 and Figure 2). In addition, we analyzed the activity level at which the R time of KL first exceeded the upper limit of the established reference range (Figure 3).

Factor II (FII): Even when factor activity was reduced to 4.6%, the R time for the three activators showed no significant prolongation compared to the baseline at an activity level of 91% (Table 5). Moreover, at the 4.6% factor activity level, the R time of KL remained within the reference range of KL (Figure 3A). No significant differences in sensitivity were observed among the three activators, either in overall comparisons or at each individual factor activity level (Figure 1A and Figure 2A).

Factor V (FV): Relative to the 85.0% activity level, significant R prolongation of KL first appeared at 42.5% activity and persisted across all lower tested activity levels down to 4.3%. For EA, significant differences were observed continuously at factor activity levels below 25.5%. For CS, significant R prolongation occurred only at isolated FV points (4.3%, 17.0%, 42.5%), with R tending to rise as FV activity decreased without continuous significance(Table 5). At activities of 25.5%, R time of KL exceeded the upper reference limit (Figure 3B). For overall SDFD comparisons, KL significantly outperformed EA (mean difference 18.35%, 95% CI 14.46–22.25%) and CS (6.886%, 0.6178–13.15%), while EA was significantly lower than CS (−11.47%, −17.35–−5.584%) (Figure 1B). At stratified activity levels, the SDFD of KL was significantly higher than EA at 8.5%, 17.0%, 34.0% and 42.5% activity; the SDFD of EA was significantly lower than CS at 42.5% activity (Figure 2B).

Factor VIII (FVIII): Relative to the 84.0% activity level, KL exhibited the first significantly prolonged R time at 25.2% activity and persisted across all lower tested activity levels down to 4.2%. EA exhibited significant R time prolongation at 8.4%, 25.2%, and 33.6% activity levels, but not at 4.2% or 16.8%. For CS, significant R prolongation was only detected at 4.2% (Table 5). Despite these significant changes, R time for KL stayed within the reference range (Figure 3C). In the overall comparison of SDFD, significant differences were found in KL and EA compared with CS (KL vs. CS: mean difference 12.96%, 95% CI 6.194–19.73%; EA vs. CS: 11.26%, 95% CI 4.554–17.97%) (Figure 1C). At the 4.2% activity level, both KL and EA had significantly higher SDFD than CS, and KL had significantly higher SDFD than EA (Figure 2C).

Factor IX (FIX): For KL, significant R prolongation first occurred at 25.2% activity and persisted at all lower levels down to 4.2%. EA showed discontinuous significant R prolongation at 4.2%, 16.8%, 33.6%, and 50.4%. No significant R prolongation was found for CS, with no consistent rise in R alongside reduced FIX activity (Table 5). The R time of KL exceeded the upper reference limit only when activity dropped to 4.2% (Figure 3D). For overall SDFD comparisons, KL and EA significantly outperformed CS (KL vs. CS: mean difference 23.41%, 95% CI 14.84–31.99%; EA vs. CS: 19.32%, 95% CI 12.49–26.16%) (Figure 1D). Compared with CS, both KL and EA exhibited higher SDFD across FIX activity levels; significant differences were observed between KL and CS at 4.2%, 16.8%, and 50.4%, and between EA and CS at 50.4% (Figure 2D).

Factor X (FX): For KL, significant R prolongation first occurred at 9.2% and 4.6% activity levels. EA had discontinuous significant R prolongation at 18.4% and 4.6%. CS showed significant R prolongation only at 9.2% (Table 5). The R time of KL exceeded the reference range only at the lowest tested activity of 4.6% (Figure 3E). KL and EA were significantly superior to CS in overall SDFD comparisons (KL vs. CS: mean difference 9.781%, 95% CI 0.4436–19.12%; EA vs. CS: 8.010%, 95% CI 0.5882–15.43%) (Figure 1E). No significant differences in SDFD among KL, EA, and CS were found at any FX activity level (Figure 2E).

Factor XI (FXI): KL developed significant R prolongation starting at 18.4% FXI activity and maintained significance at 9.2% and 4.6%. EA showed scattered significant R prolongation at 9.2% and 4.6%. CS had no significant R prolongation at any activity level and lacked a consistent rising trend of R with reduced activity (Table 5). The R time for KL exceeded the reference range at 4.6% activity (Figure 3F). Overall SDFD analysis showed that KL and EA had significantly higher SDFD values than CS (KL vs. CS: mean difference = 12.96%, 95% CI 6.194–19.73%; EA vs. CS: mean difference = 11.26%, 95% CI 4.554–17.97%) (Figure 1F). At the 4.6% activity level, SDFD values of KL and EA were significantly higher than those of CS; KL also had a significantly higher SDFD value than CS (Figure 2F).

Factor XII (FXII): Relative to the highest activity, KL and EA both first showed significant R-time prolongation at 40.4% activity, persisting down to 5.1%. CS achieved significant R prolongation only at 40.4% and 20.2%, with no consistent increase in R accompanying reduced activity (Table 5). Even at the lowest factor activity level, the R-time for KL did not exceed the upper limit of the reference range (Figure 3G). Overall SDFD analysis showed that KL had significantly lower SDFD values than EA (mean difference = −15.56%, 95% CI −21.00–−10.11%), whereas EA had significantly higher SDFD values than CS (mean difference = 21.60%, 95% CI 12.55–30.66%) (Figure 1G). Stratified analysis revealed that EA had significantly higher SDFD than KL at 10.1%, 20.2%, 30.3%, and 60.6% activity levels, and also higher SDFD than CS at 20.2% and 40.4% (Figure 2G).

## 4. Discussion

This study evaluated and compared the sensitivity of three contact activators—KL, EA, and CS—for detecting single coagulation factor deficiencies in reconstituted whole-blood samples using TEG. Our first approach to assessing the factor sensitivities of the three activators was to determine the factor activity level at which a statistically significant prolongation of R-time first occurred. We also introduced a novel method, SDFD, enabling this comparison without establishing reference ranges. The results showed a clear, factor-specific difference in how sensitive each activator was.

At the concentrations tested in this study, KL showed the highest sensitivity for FV, FVIII, FIX and FXI; it could detect significant R time prolongations at milder deficiency levels that persisted down to the lowest activity level (e.g., FV at 42.5–4.6%, FVIII at 25.2–4.2%, FXI at 18.4–4.6%). EA showed intermediate to high sensitivity and particular superiority for FXII deficiency. Except for FV, CS consistently showed the lowest sensitivity and often required severe deficiency (≤10% activity) to produce a significant change in R times. Notably, for KL, the factor activity threshold required to exceed the clinical reference range was consistently lower than that for achieving statistical significance.

In the present study, whether assessed overall or at individual factor activity levels, sensitivity to FII deficiency was uniformly low across all activators, with no significant differences observed—a finding consistent with prior reports on APTT reagent sensitivity [20]. For FV, overall comparison showed that KL showed the highest sensitivity, followed by CS, while EA showed the lowest, with KL significantly higher SDFD than EA at 42.5%, 34.0%, 17.0%, and 8.5% activity levels. This contrasts with a previous APTT study where colloidal silica exhibited superior FV sensitivity to kaolin [20]. Both KL and EA had significantly higher mean SDFD than CS for FVIII. At 4.2% activity, both remained significantly more sensitive than CS, and KL significantly outperformed EA. Traditional APTT comparisons report that ellagic acid and micronized silica possess comparable, superior FVIII sensitivity to kaolin, while another study found no difference between kaolin and colloidal silica [20,21,22]. For FIX, both KL and EA were significantly more sensitive than CS in the overall SDFD comparison, and at 50.4%, 16.8%, and 4.2% activity levels, KL showed significantly higher SDFD than CS but did not differ significantly from EA. This contradicts APTT studies where kaolin consistently showed the lowest sensitivity [20,21,22]. In the overall comparison for FX, KL and EA exhibited comparable sensitivity, both of which were higher than that of CS, whereas an earlier APTT study found ellagic acid and micronized silica more sensitive than kaolin [21]. However, no significant differences were observed among the three activators at individual activity levels. Notably, FX sensitivity among colloidal silica-based APTT reagents is variable [20]. For FXI, KL and EA both exhibited significantly higher mean SDFD than CS. At 4.2% activity, KL exhibited significantly greater sensitivity than both EA and CS. FXII was the only factor for which EA showed higher sensitivity than KL, both in overall comparisons and at individual factor activity levels, aligning with traditional APTT findings [21]. These differences from previous APTT findings likely arise from two factors. First, APTT uses plasma, whereas TEG uses whole blood containing platelets and RBCs that contribute to coagulation. Second, in whole blood, cellular components may interact with activators and their downstream effects in ways absent in plasma.

Several observations in this study invite mechanistic interpretation. The explanations proposed below remain speculative, however, and are not directly shown by our data. FII deficiency showed uniformly low sensitivity across activators, which may be explained by FII’s position at the terminal common pathway: when FII activity becomes rate-limiting, upstream differences in activator efficiency could be compressed by this shared bottleneck, potentially converging the performance of all activators. This possibility, however, remains to be tested in future mechanistic studies. Of note, KL and EA showed divergent sensitivity profiles for FV and FXII. One possible interpretation is that KL may modulate FV-mediated thrombin generation, but this remains speculative and requires further investigation. Nevertheless, our present data cannot confirm or exclude this possibility. Beyond these factor-specific observations, the differential sensitivity among activators may also reflect differences in their physicochemical properties. Kaolin, ellagic acid, and silica each activate the intrinsic coagulation pathway via negative charges on their particle surfaces; however, their sensitivities to coagulation factor deficiencies vary considerably. Early studies have suggested that the activation efficiency of FXII in plasma is related to the surface energy of the activator (glass particles), with higher surface energy leading to higher activation efficiency [34,35]. Another study suggested that silica nanoparticle curvature may be an important determinant of coagulation activity, with nanoparticles of lower surface curvature exhibiting higher coagulation activity [36]. Therefore, the differences in sensitivity among different activators could be attributable to variations in their surface energy and particle curvature. Such differences may contribute to inconsistent thrombin generation efficiency at different factor concentrations, potentially resulting in sensitivity differences. Furthermore, previous studies have shown that the binding of Kaolin to FXII is specific via the epidermal growth factor-1 domain (EGF-1 domain) of FXII, and targeting these binding sites significantly prolongs coagulation time [37]. Therefore, another mechanism for the differential sensitivity among activators could be unanticipated inhibition or synergism of certain activators with specific serine proteases or with the aforementioned non-enzymatic coagulation factors. Elucidating which physicochemical characteristics of activators determine the differential sensitivity to factor deficiencies is an important direction for our future studies.

Our findings indicate that kaolin provides the highest sensitivity for detecting deficiencies in most intrinsic and common pathway factors in the thromboelastography detection system. When KL is used, R time shows statistically significant prolongation for most factors as activity falls within 18.0–42.0%, yet typically remains within the clinical reference range (3.9–8.3 min). For instance, R time prolongation became significant at FIX and FXI activity levels of 25.2% and 18.4%, respectively, yet exceeded the upper limit of the reference range only when activity dropped to 4.2% and 4.6%, respectively. These observations align with findings from several clinical studies. In a study investigating R time for predicting coagulation factor deficiency in trauma patients, even when the INR (international normalized ratio) exceeded 2.0 (corresponding to approximately 30% factor activity), 95.7% of R time values remained within the provided clinical reference range. However, R time was significantly prolonged in patients with INR > 1.5 (approximately 50% activity) compared to those with INR < 1.5 (3.2 [2.7–3.8] vs. 4.7 [3.7–5.6] minutes, *p* < 0.001) [11]. Similarly, Nascimento, B. et al. reported that R time showed very low sensitivity (33%) for detecting vitamin K-dependent coagulation factor deficiency [16]. Consequently, using R time exceeding the reference range as the sole diagnostic criterion for factor deficiency is insufficiently rigorous, underscoring the need for more stringent, context-specific ranges or more sensitive TEG reagents.

The sensitivity of APTT reagents is related to factors such as the type of surface activator used, the source and concentration of phospholipids [38]. However, besides the type of surface activator, other factors may also affect TEG reagent factor sensitivity. These factors require further investigation, which is a crucial step for developing highly sensitive reagents. During the development of TEG reagents, any change in the composition or concentration of the reagent would require re-establishment or validation of reference ranges for sensitivity evaluation, following the principles of CLSI H47-A2 for APTT reagents. This will consume substantial manpower, material resources, and time. Moreover, the sample for TEG testing is whole blood, whereas the CLSI H47-A2 employs plasma, which may lead to a matrix effect. The SDFD method used in this study enables relative sensitivity comparison during early reagent development without requiring reagent-specific reference ranges, providing a practical tool for activator screening prior to final clinical sensitivity validation using reference ranges. This may simplify the early-stage development process and reduce the time and cost required for activator screening. Although the reconstituted whole-blood samples used in this study remain an in vitro model, they partially mimic the cellular composition of native whole blood and may thereby reduce the matrix effect.

Several limitations of this study should be acknowledged. First, the reconstituted whole-blood model is an in vitro system that cannot fully replicate in vivo conditions (e.g., endothelium, inflammation, inhibitors, drugs) and represents only single-factor deficiency, whereas clinical coagulopathies typically involve multiple factors. These limitations restrict direct clinical extrapolation. Moreover, for some factors, differences at individual activity levels were not consistently significant, likely due to the small sample size (*n* = 3) and reduced power after correction, which may weaken the reliability of stratified comparisons and limit the strength of conclusions at those levels. Second, the reagents contained no phospholipids, so the findings may not apply to phospholipid-containing formulations. Third, platelet/RBC characteristics and the use of R time as the primary criterion may not fully capture activator sensitivity, potentially leading to misleading interpretation and weakening conclusions. Moreover, SDFD has not been validated as a clinical diagnostic sensitivity measure, as it lacks validation against clinical classification, ROC analysis, or independent datasets, so its reliability remains unestablished.

To address these limitations, future work should move in three directions. First, larger and more clinically relevant samples—such as patient-derived or multi-factor deficiency plasma—are needed to confirm the present findings and to assess the impact of phospholipids [39]. Second, concentration-response studies combined with physicochemical characterization (e.g., surface charge, particle size, Zeta potential, and hydrophilicity/hydrophobicity) could reveal which properties drive activator sensitivity [34,35,36]. Third, SDFD should be compared with established sensitivity metrics (e.g., regression slopes and fitted thresholds), and further tested in actual reagent development to establish reagent-specific reference ranges [11,20,40].

## 5. Conclusions

In conclusion, this study compared the relative sensitivity of three contact activators used in TEG for detecting coagulation factor deficiencies. Using two relative analytical metrics—the factor activity threshold for the first statistically significant R time prolongation and the SDFD-based comparison—it showed that this relative sensitivity depends on both the activator type and the specific factor assessed. This activator-dependent pattern in reconstituted whole-blood samples is not consistent with the sensitivity responses established in most plasma-based APTT testing. At the concentrations tested in this study, kaolin exhibited the highest overall sensitivity for screening most intrinsic and common pathway coagulation factor deficiencies. Under the same experimental conditions, ellagic acid showed the highest sensitivity for detecting FXII deficiency. Conversely, colloidal silica showed the lowest sensitivity for early deficiency detection, except for FV. Furthermore, for KL, the R time exceeded the reference range at a much lower factor activity than was required for statistically significant prolongation, suggesting that this reference range may not be rigorous enough. Future research should focus on developing TEG reagents with higher sensitivity using the SDFD method and on establishing more appropriate reference ranges. It should be noted that SDFD remains an operational relative metric and has not yet been validated as a clinical diagnostic sensitivity measure.

## Figures and Tables

**Table 1:** Mixing ratios of platelet-rich factor-deficient plasma (PRDP), platelet-rich standard plasma (PRSP), and red blood cells (RBCs) for reconstitution of different factor activities.

Components	Mixing Ratio							
PRDP	19	18	16	14	12	10	8	0
PRSP	1	2	4	6	8	10	12	20
RBCs	20	20	20	20	20	20	20	20

**Table 2:** Performance evaluation of Ellagic acid-based activators (EA) and Silica-based activators (CS).

Reagents	R			Angle			MA		
	RAD of EA & Kaolin-Based Activators (KL) and CS & KL								
	Batch1	Bacth2	Bacth3	Batch1	Batch2	Batch3	Batch1	Batch2	Batch3
EA	3.10%	1.10%	6.30%	0.1%	2.8%	3.3%	1.1%	3.1%	1.7%
CS	9.40%	10.30%	2.60%	8.0%	4.8%	4.5%	5.4%	2.9%	5.5%
	Repeatability								
EA	7.78%	6.79%	6.12%	2.03%	1.26%	1.05%	5.87%	5.07%	8.89%
CS	8.73%	7.79%	9.52%	2.45%	1.88%	1.76%	5.67%	4.17%	8.13%
	Batch-to-batch variation								
EA	3.21%			0.87%			3.95%		
CS	9.18%			0.41%			3.45%		

Performance evaluation was conducted on three consecutive batches of EA and CS.

**Table 3:** Platelet Counting in platelet-rich standard plasma (PRSP).

Batch	The Number of Platelets in the PRSP (/mL)						
	FII	FV	FVIII	FIX	FX	FXI	FXII
1	1.30 ± 0.19 × 10^8^	1.47 ± 0.43 × 10^8^	1.81 ± 0.06 × 10^8^	2.14 ± 0.15 × 10^8^	1.35 ± 0.03 × 10^8^	2.12 ± 0.11 × 10^8^	1.84 ± 0.15 × 10^8^
2	1.22 ± 0.30 × 10^8^	1.58 ± 0.06 × 10^8^	2.10 ± 0.11 × 10^8^	2.27 ± 0.13 × 10^8^	1.48 ± 0.26 × 10^8^	2.22 ± 0.09 × 10^8^	2.03 ± 0.11 × 10^8^
3	1.36 ± 0.17 × 10^8^	1.38 ± 0.24 × 10^8^	1.72 ± 0.09 × 10^8^	2.43 ± 0.13 × 10^8^	1.34 ± 0.19 × 10^8^	1.87 ± 0.15 × 10^8^	1.78 ± 0.31 × 10^8^

Values are expressed as the mean ± SD (*n* = 3).

**Table 4:** Platelet function after washing and resuspension.

Sample	The MA of Reconstituted Whole-Blood Samples (RWB)/Standard Human Plasma (SHP) with 100% Factor Activity (mm)						
	FII	FV	FVIII	FIX	FX	FXI	FXII
RWB	51.03 ± 0.81	47.03 ± 0.76	52.6 ± 1.51	57.73 ± 1.53	49.73 ± 0.15	57.73 ± 1.53	56.83 ± 0.8
SHP	29.1 ± 1.95	29.1 ± 1.95	29.1 ± 1.95	29.1 ± 1.95	29.1 ± 1.95	29.1 ± 1.95	29.1 ± 1.95

Data are mean ± SD; reconstituted whole-blood samples: *n* = 3 independent runs; standard plasma: triplicate technical replicates from a single lot, with no formal statistical comparison performed.

**Table 5:** R time of various Factor activities of reconstituted whole-blood samples measured with different activators.

Factor	Reagents	R Time (min)							
		Factor Activities of Reconstituted Whole-Blood Samples							
FII		4.6%	9.1%	18.2%	27.3%	36.4%	45.5%	54.6%	91.0%
	KL	6.80 ± 0.00	6.23 ± 0.31	6.03 ± 0.21	6.30 ± 0.10	5.90 ± 0.17	5.77 ± 0.15	5.60 ± 0.10	6.10 ± 0.26
	EA	6.67 ± 0.21	6.20 ± 0.17	6.17 ± 0.45	6.27 ± 0.61	6.10 ± 0.17	6.60 ± 0.35	6.07 ± 0.12	6.20 ± 0.10
	CS	6.33 ± 0.47	5.97 ± 0.57	6.13 ± 0.06	6.37 ± 0.15	6.40 ± 0.26	6.63 ± 0.65	6.10 ± 0.53	6.30 ± 0.10
FV		4.3%	8.5%	17.0%	25.5%	34.0%	42.5%	51.0%	85.0%
	KL	9.27 ± 0.59 *	8.73 ± 0.64 *	8.37 ± 0.49 *	8.47 ± 0.55 *	8.10 ± 0.36 *	7.70 ± 0.44 *	7.03 ± 0.31	5.80 ± 0.10
	EA	7.53 ± 0.67 *	7.47 ± 0.12 *	7.10 ± 0.17 *	7.03 ± 0.40 *	6.63 ± 0.12	6.20 ± 0.17	6.17 ± 0.29 *	5.57 ± 0.38
	CS	9.13 ± 0.38 *	8.97 ± 0.35	8.97 ± 0.50 *	8.43 ± 0.21	8.47 ± 0.76	8.00 ± 0.52 *	7.40 ± 0.79	6.30 ± 0.50
FVIII		4.2%	8.4%	16.8%	25.2%	33.6%	42.0%	50.4%	84.0%
	KL	7.87 ± 0.15 *	7.43 ± 0.12 *	6.83 ± 0.12 **	6.57 ± 0.21 **	6.63 ± 0.06	5.93 ± 0.25	6.10 ± 0.10	5.70 ± 0.20
	EA	8.13 ± 0.59	7.23 ± 0.25 *	6.97 ± 0.23	6.80 ± 0.10 **	6.57 ± 0.12 *	5.93 ± 0.06	6.13 ± 0.06	6.07 ± 0.06
	CS	8.00 ± 0.20 *	7.40 ± 0.61	7.30 ± 0.46	7.33 ± 0.85	7.23 ± 0.58	6.97 ± 0.59	7.27 ± 0.12	6.63 ± 0.35
FIX		4.2%	8.4%	16.8%	25.2%	33.6%	42.0%	50.4%	84.0%
	KL	8.80 ± 0.53 *	8.03 ± 0.12 **	7.07 ± 0.15 **	6.67 ± 0.23 *	6.30 ± 0.20	5.80 ± 0.17	5.90 ± 0.35	5.53 ± 0.15
	EA	8.50 ± 0.00 *	8.13 ± 0.23	7.43 ± 0.47 *	6.93 ± 0.51	6.63 ± 0.47 **	6.37 ± 0.58	6.57 ± 0.51 ****	5.97 ± 0.51
	CS	7.10 ± 0.36	7.20 ± 0.53	6.77 ± 0.84	6.57 ± 0.21	6.03 ± 0.90	6.53 ± 0.12	5.93 ± 0.21	6.47 ± 0.35
FX		4.6%	9.2%	18.4%	27.6%	36.8%	46.0%	55.2%	92.0%
	KL	9.57 ± 0.49 *	8.07 ± 0.06 **	7.37 ± 0.31	7.87 ± 0.42	7.17 ± 0.25	6.73 ± 0.25	6.87 ± 0.31	6.50 ± 0.10
	EA	8.67 ± 0.32 *	7.90 ± 0.66	7.87 ± 0.15 **	7.67 ± 0.15	7.50 ± 0.69	6.77 ± 0.23	7.00 ± 0.26	6.57 ± 0.25
	CS	8.67 ± 0.32	8.13 ± 0.55 *	7.93 ± 0.80	7.23 ± 0.50	7.70 ± 0.60	7.87 ± 0.31	6.90 ± 0.26	7.23 ± 0.67
FXI		4.6%	9.2%	18.4%	27.6%	36.8%	46.0%	55.2%	92.0%
	KL	8.70 ± 0.35 **	7.93 ± 0.40 *	7.53 ± 0.06 *	7.23 ± 0.31	6.93 ± 0.45	6.37 ± 0.15	6.67 ± 0.12	6.17 ± 0.12
	EA	8.80 ± 0.10 **	8.37 ± 0.29 *	7.53 ± 0.51	7.60 ± 0.69 *	7.27 ± 0.51	6.40 ± 0.36	6.60 ± 0.20	6.40 ± 0.10
	CS	6.13 ± 0.15	5.70 ± 0.44	5.93 ± 0.38	6.27 ± 0.23	5.90 ± 0.61	5.37 ± 0.42	5.50 ± 0.40	5.50 ± 0.26
FXII		5.1%	10.1%	20.2%	30.3%	40.4%	50.5%	60.6%	101.0%
	KL	8.10 ± 0.56 *	7.23 ± 0.31 *	7.17 ± 0.40 *	6.60 ± 0.10 **	6.53 ± 0.25 *	5.80 ± 0.20	5.77 ± 0.23	5.50 ± 0.10
	EA	8.33 ± 0.42 **	7.80 ± 0.40 **	6.83 ± 0.15 **	6.53 ± 0.31 **	6.23 ± 0.15 ****	5.37 ± 0.25	5.63 ± 0.15	4.83 ± 0.15
	CS	8.40 ± 0.70	8.60 ± 0.78	7.90 ± 0.17 **	8.30 ± 0.46	7.87 ± 0.21 *	6.50 ± 0.60	7.07 ± 0.58	6.70 ± 0.10

Values are expressed as the mean ± SD (*n* = 3 independent experiments). A one-way repeated-measures mixed-effects model with experimental batch as a random intercept was used, followed by Dunnett’s post-hoc test comparing R of each factor activity to the R of the highest activity as the control. * *p* < 0.05; ** *p* < 0.01; **** *p* < 0.0001 vs. R of highest factor activity.

## Data Availability

The raw data supporting the conclusions of this article will be made available by the authors on request.

## Abbreviations

The following abbreviations are used in this manuscript:

FII	Coagulation factor II
FV	Coagulation factor V
FVIII	Coagulation factor VIII
FIX	Coagulation factor IX
FX	Coagulation factor X
FXI	Coagulation factor XI
FXII	Coagulation factor XII
APTT	Activated partial thromboplastin time
PT	Prothrombin time
TEG	Thromboelastography
R	Reaction time
K	K time
MA	Maximum amplitude
KL	Kaolin-based activators
EA	Ellagic acid-based activators
CS	Silica-based activators
PRP	Platelet-rich plasma
PRDP	Platelet-rich factor-deficient plasma
PRSP	Platelet-rich standard plasma
RBCs	Red blood cells
SDFD	Sensitivity for detecting factor deficiencies
RSD	Relative average deviations
